# Episodic Headache, Periorbital Pain, and Multifocal Paresthesias as Presenting Symptoms of Central Neurocytoma: A Case Report

**DOI:** 10.7759/cureus.35334

**Published:** 2023-02-22

**Authors:** Permphan Dharmasaroja

**Affiliations:** 1 Chakri Naruebodindra Medical Institute, Faculty of Medicine Ramathibodi Hospital, Mahidol University, Samut Prakan, THA

**Keywords:** swi, dwi, brain tumor, midline shift, unilateral headache, intracranial pressure, obstructive hydrocephalus, central neurocytoma

## Abstract

Central neurocytoma (CN) is a rare intraventricular tumor. The common presenting symptoms of CN are headache, vomiting, and visual disturbance, which results from increased intracranial pressure. This report presents a case of CN with unusual clinical presentations. A 25-year-old female with CN presented with a one-day history of unilateral headache, ipsilateral periorbital pain, multifocal paresthesias, and vomiting. Magnetic resonance images showed an intraventricular mass with a soap-bubble appearance and numerous cystic areas typical for CN, causing obstructive hydrocephalus and a midline shift. After one night of rest, her headache, periorbital pain, and paresthesias disappeared. It is possible that the tumor could be mobile with regard to the patient's head position, causing occasional obstruction of the foramen of Monro. Due to the tumor size, which was larger than 4 centimeters, the surgical approach with either gross tumor resection or subtotal resection plus adjuvant radiotherapy should be carefully considered.

## Introduction

Central neurocytoma (CN) is a rare, benign, intraventricular tumor with potential malignancy, with an annual incidence rate of about 0.032 per 100,000 population and a peak incidence in the 20-34 years of age [1]. It contributes to less than 0.5% of all brain tumors [2]. The most common presenting symptom of CN is headache, which stems from increased intracranial pressure (ICP) [3-7]. Other reported symptoms include nausea and vomiting, visual disturbance, weakness, paresthesias, balance problems, seizure, memory impairment, aphasia, and tinnitus [3,5-7]. However, CN may be asymptomatic and found as incidental imaging findings [3,7].

Patients with increased ICP typically present with headache, blurred vision, and vomiting. Headaches are frequently reported as having a generalized throbbing or bursting quality, worsening in the morning, and made worse by lying down, coughing, or sneezing [8]. The blurring of vision is caused by papilledema, a reliable sign of increased ICP. However, patients with acute increased ICP do not usually show papilledema because it needs several days of elevated ICP to develop. Sensory symptoms, such as paresthesias, are not common in CN, and are possibly related to the lesions in the parietal lobe. The author presents the case of a young patient, presenting with episodic unilateral headache, multifocal paresthesias, and vomiting, radiologically diagnosed with CN causing obstructive hydrocephalus.

## Case presentation

A 25-year-old female presented with a one-day history of an acute onset of left temporal headache radiating from the left periorbital area. The headache was constant and pressing-like in quality, which was not relieved by paracetamol. The patient vomited once without nausea. By the morning of coming to the hospital, the headache had subsided but it occurred when her head was shaken. The periorbital pain remained and the patient reported experiencing tingling sensations in her right cheek and left palm. The patient stated she had normal vision and no other symptoms. With careful history taking, the patient reported that she had experienced intermittent, mild headaches for three months, occurring once a week and alleviated by over-the-counter medicines and rest. She had no notable past or family history, and she did not take any medication on a regular basis. On physical examination, blood pressure was 126/90 mmHg, heart rate was 80/minute, afebrile, there were no papilledema and motor weakness, and otherwise was normal.

**Figure 1:**
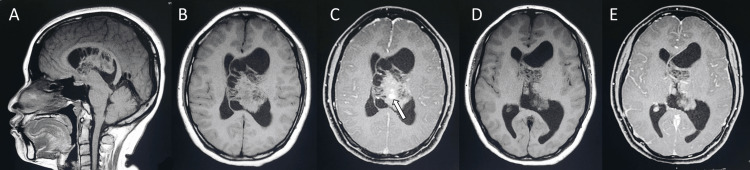
Magnetic resonance T1WI of the brain. (A) Midsagittal plane; (B) Axial plane at the lateral ventricle level; (C) Axial plane with gadolinium at the lateral ventricle level; (D) Axial plane at the third ventricle level; (E) Axial plane with gadolinium at the third ventricle level. The mass is isointense with mild to moderate heterogeneous enhancement (arrow). Obstructive hydrocephalus and pressure effects on the left thalamus are prominent. T1WI: T1-weighted image

**Figure 2:**
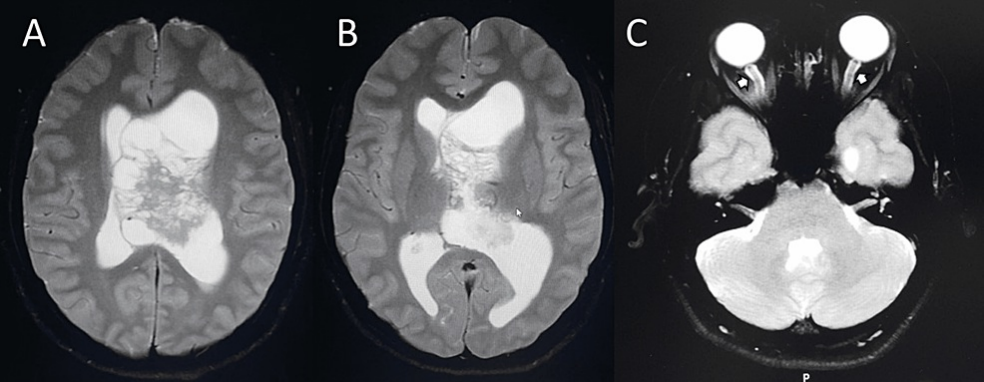
Magnetic resonance T2WI of the brain. (A) Axial plane at the lateral ventricle level; (B) Axial plane at the third ventricle level. The mass is isointense with a soap-bubble appearance and numerous cystic areas; (C) Mild distension of the subarachnoid space around bilateral optic nerves (arrows). T2WI: T2-weighted image

As the patient’s unusual headache, periorbital pain, and multifocal paresthesias suggested intracranial lesions, the patient was admitted and a brain magnetic resonance imaging (MRI) was performed. T1 and T2-weighted images revealed a large heterogeneously enhancing solid-cystic intraventricular mass that involved the body of bilateral lateral ventricles (more on the left), frontal horn, trigone of the left lateral ventricle, and septum pellucidum, obstructing bilateral foramen of Monro and causing obstructive hydrocephalus as well as pressure effects on the left basal ganglia and left thalamus (Figures 1, 2). The mass was measured to have anteroposterior, transverse, and vertical dimensions of around 6.4 x 4.5 x 4.2 centimeters. Mild prominent subarachnoid space around bilateral optic nerves was seen (Figure 2C).

**Figure 3:**
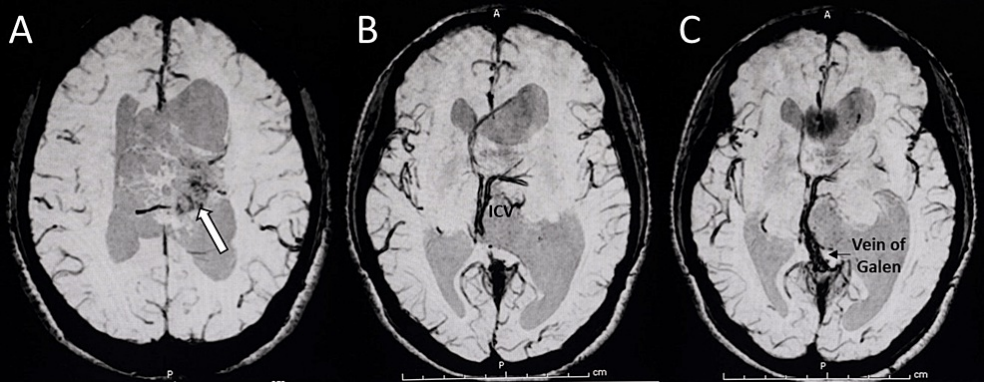
Axial SWI of the brain. (A) Hypointense blooming in the solid portion of the mass indicates calcifications (arrow); (B-C) A midline shift of 1.5-centimeters to the right and right deviation of bilateral internal cerebral veins (ICV) and the vein of Galen were noted. SWI: susceptibility-weighted imaging; ICV: internal cerebral vein

**Figure 4:**
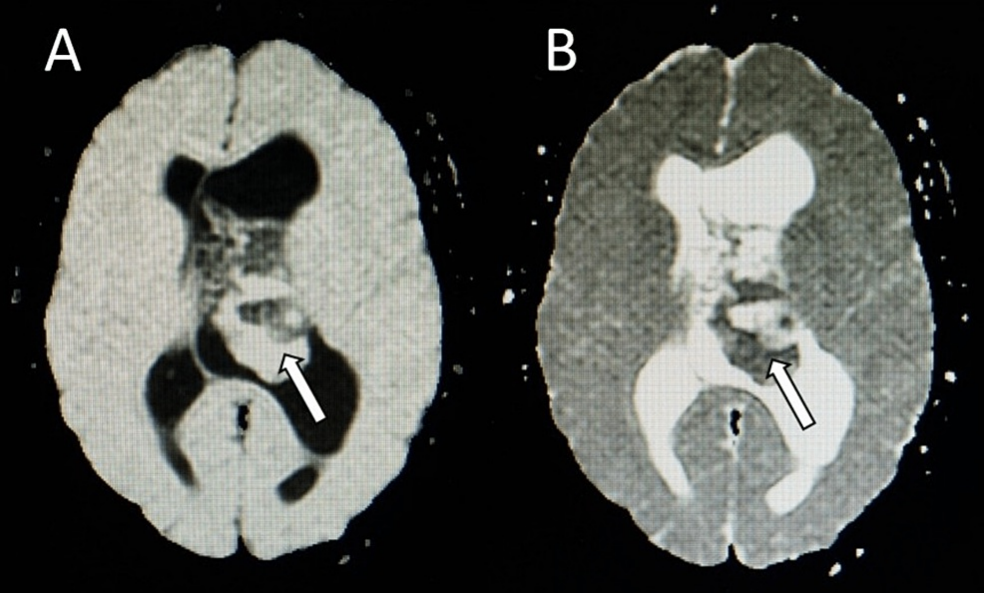
Axial DWI of the brain. (A) DWI and (B) enhanced or exponential DWI shows diffusion restriction of the solid portion of the mass (arrows), suggesting high cellularity of the mass. DWI: diffusion-weighted imaging

Susceptibility-weighted imaging (SWI) revealed hypointense blooming suggestive of internal calcifications in the solid portion of the mass (Figure 3A). A 1.5-centimeters midline shift to the right and right deviation of the vein of Galen and bilateral internal cerebral veins were seen (Figures 3B, 3C). Diffusion-weighted imaging (DWI) showed diffusion restriction in the solid portion (Figure 4). These radiological findings were suggestive of central neurocytoma.

After one night of admission, the patient was referred to a tertiary hospital for surgical management. On that day before referral, the patient stated that she awakened normally and had neither headache nor periorbital pain. Her paresthesias also disappeared.

## Discussion

Episodic headaches, which the patient had experienced once a week for three months, may prevent the patient from seeking medical attention. However, the recent episode of unusual headache with periorbital area involvement and multifocal paresthesias suggests that the disease is progressive and should be taken seriously. Unilateral headache and radiating periorbital pain as present in this patient are uncommon symptoms of increased ICP. Classical worsening of headache in the morning is also not present in this patient. For periorbital involvement, retro-orbital pain has been reported as an associated symptom of idiopathic intracranial hypertension [9]. Since the optic nerve sheath is an anatomical extension of the dura mater surrounding the nerve, it is known that elevated ICP can produce distension of the subarachnoid space around the optic nerve, which may contribute to increased intraocular pressure and cause retro- or periorbital pain [10]. The on-and-off nature of the patient's headaches raises the possibility that the portion of the tumor blocking the foramen of Monro may be mobile with regard to the patient's head position.

Multifocal paresthesias present in this patient may reflect thalamic involvement, as shown by the pressure effect in the MRI. As the venous system from the thalamus drains to internal cerebral veins, which originate at the foramen of Monro, the patient's paresthesias may be caused in part by the deviation of the bilateral internal cerebral veins. Both the pressure effect and deviated veins might affect the tissue pressure in the thalamus, generating paresthesias on both sides of the body. Theoretical pathophysiology for these paresthesias includes central imbalance, central disinhibition, central sensitization, and the inflammatory response of the intra-thalamic neural pathway [11].

The anterior half of the lateral ventricle is the most common location for a CN in the supratentorial ventricular system, and 26% of CN extends into the third ventricle [6]. Based on radiological images, other tumors can be excluded in this patient. Astrocytomas and ependymomas often lack intratumoral cysts and calcifications. For oligodendroglioma, intratumoral calcifications are frequently extensive and irregular. MR findings shown in this patient are typical for CN, which reveals a tumor that is isointense in T1-weighted images with mild to moderate heterogeneous enhancement, a soap-bubble appearance and numerous cystic areas in T2-weighted images, calcified areas in SWI, and diffusion restriction of the solid portion in DWI.

The tumor size of CN in this patient is rather big. A recent retrospective analysis of 413 patients revealed that the survival rate was poorer for tumors greater than 4 centimeters than for smaller tumors [12]. Gross total resection (GTR), if feasible, has been the cornerstone of early therapy for CN, and it is a favorable factor affecting the prognosis of CN. If subtotal resection (STR) is more suitable, postoperative radiotherapy has been shown to improve survival in these patients [13].

## Conclusions

Although CN can be present with symptoms and signs of increased ICP, a physician should be aware of its unusual presentations. Intermittent obstruction of the ventricular system by the tumor may not result in papilledema. Episodic headaches and vomiting without nausea may be the only warning symptoms of CN, which indicates intermittent increased ICP. Other accompanying symptoms may be useful to urge conducting investigation. In the present case, multifocal paresthesias may indicate thalamic pathology. Considering all of these symptoms together, it may point to an intraventricular lesion, especially at the third ventricle, where the thalamus forms the walls of the chamber. The diagnosis of CN can be supported by radiological findings, and if possible, should be confirmed by histological and immunological studies. Gross total resection is the preferred treatment and favors a good prognosis. In case of a tumor size larger than 4 centimeters and complete removal of the tumor is not possible, radiotherapy following subtotal resection should be the treatment of choice.
